# Comparative evaluation of spinal versus epidural anesthesia in lower limb orthopedic surgery: Hemodynamic stability and block duration

**DOI:** 10.6026/973206300223707

**Published:** 2026-06-30

**Authors:** Neetesh Gautam, Narayan Hari Sharma, Irfan Khan

**Affiliations:** 1 Department of Anaesthesiology, Gajra Raja Medical College, Gwalior, Madhya Pradesh, India; 2 Department of Anaesthesiology, Janak Hospital, Gwalior, Madhya Pradesh, India

**Keywords:** Spinal anesthesia, epidural anesthesia, hemodynamic stability, orthopedic surgery, block duration, post-operative analgesia

## Abstract

Regional anesthesia remains essential in lower limb orthopedic surgeries for providing effective intraoperative anesthetic control and post-operative analgesia with fewer systemic side effects. Therefore, it is of interest to evaluate the fifty patients undergoing elective lower limb orthopedic procedures to assess spinal versus epidural anesthesia in relation to hemodynamic stability and block duration. Twenty-five patients received hyperbaric bupivacaine via spinal route and twenty-five received bupivacaine through a lumbar epidural catheter. Spinal anesthesia demonstrated faster onset and denser surgical block but was associated with higher incidence of hypotension and bradycardia. Epidural anesthesia produced slower onset yet maintained better hemodynamic stability along with prolonged post-operative analgesia. Thus, data shows the both techniques are safe and effective, with choice guided by surgical duration, patient profile and post-operative pain management requirements.

## Background:

Regional anesthesia has several benefits over general anesthetics that make it a popular choice for lower limb orthopedic procedures. These include better post-operative analgesia, less blood loss, less pulmonary morbidity and quicker mobilization, among other things. When it comes to orthopedic treatments, the two most common neuraxial blocks are spinal and epidural anesthesia [1]. A local anesthetic is injected into the subarachnoid region during spinal anesthesia, quickly blocking dense sensation and motor functions. For many lower limb procedures, this approach is the favored choice because to its technical simplicity, reliability and cost-effectiveness [2]. However, spinal anesthesia is associated with abrupt sympathetic blockade, which may lead to hypotension, bradycardia, and hemodynamic instability, particularly in patients undergoing prolonged procedures or those with limited cardiovascular reserve [3]. Epidural anesthesia, in contrast, involves administration of local anesthetic into the epidural space through a catheter, allowing graded and titratable blockade. This technique produces a slower onset of sensory and motor block compared with spinal anesthesia but offers greater control over block height and duration [4]. Epidural anesthesia is particularly advantageous in patients requiring prolonged surgery, extended post-operative analgesia, or better cardiovascular stability. Because the sympathetic blockade occurs gradually, epidural anesthesia is generally associated with fewer hemodynamic fluctuations than spinal anesthesia [5]. Hemodynamic stability is a critical determinant in selecting an anesthetic technique, especially in orthopedic patients who may be elderly, trauma-related, or have comorbid conditions such as hypertension, diabetes, or ischemic heart disease [6]. Sudden decreases in systemic vascular resistance following spinal anesthesia may lead to significant hypotension, which can compromise organ perfusion. Epidural anesthesia, by producing a more gradual sympathetic block, may reduce these risks and provide better cardiovascular control during surgery [7]. Another important factor influencing anesthetic choice is the onset and duration of sensory and motor blockade. Spinal anesthesia typically produces a faster and denser block, making it suitable for shorter procedures with predictable duration [8]. Epidural anesthesia, while slower in onset, allows continuous infusion or repeated dosing, thereby extending analgesia into the post-operative period and facilitating early rehabilitation [9]. Recent clinical research has focused on comparing these two techniques in terms of intraoperative hemodynamic stability, block characteristics, post-operative pain control and complication rates. Some studies report that spinal anesthesia provides more reliable surgical conditions, while others suggest that epidural anesthesia may be superior in maintaining cardiovascular stability and providing prolonged analgesia [10]. Therefore, it is of interest to compare the spinal and epidural anesthesia in patients having orthopedic surgery on their lower limbs.

## Materials and Methodology:

## Study design and setting:

All subjects provided written informed permission and the research was approved by the institutional ethics committee before it was performed at the Department of Anaesthesiology at a tertiary care teaching hospital.

## Study duration:

The study was carried out over a period of months.

## Sample size:

A total of 50 patients scheduled for elective lower limb orthopedic surgery under regional anesthesia were included in the study.

## Study population:

## Inclusion criteria:

[1] Patients aged 18-65 years

[2] Either gender

[3] ASA physical status I or II

[4] Scheduled for elective lower limb orthopedic procedures (fracture fixation, arthroscopy, implant removal, etc.)

[5] Willing to provide informed consent

## Exclusion criteria:

[1] Patient refusal

[2] Contraindication to regional anesthesia

[3] Coagulopathy or anticoagulant therapy

[4] Infection at puncture site

[5] Severe spinal deformity

[6] Neurological disorders

[7] Allergy to local anesthetic drugs

[8] ASA grade III or higher

## Randomization and group allocation:

Patients were randomly allocated into two groups of 25 each using computer-generated random numbers:

[1] Group S (Spinal Anesthesia, n = 25)

[2] Group E (Epidural Anesthesia, n = 25)

## Pre-operative preparation:

Before surgery, every patient had to go through pre-anesthesia testing. The evaluation included the analysis of routine investigations such as haemoglobin, blood sugar, renal function tests, electrocardiogram (ECG) and chest X-ray (where applicable). Every patient was instructed to fast for six hours before to surgery. It was common practice to connect standard monitors upon arrival in the operation room, including:

[1] Non-invasive blood pressure

[2] Pulse oximetry

[3] Electrocardiography

Baseline readings of heart rate, sap, diastolic BP, mean BP, respiratory rate and oxygen saturation were recorded. All patients were preloaded with Ringer's lactate 10 ml/kg and an 18-gauge cannula was used to ensure intravenous access.

## Anesthetic technique:

## Group S - Spinal anesthesia:

The patients were either positioned to sit or to lie on their sides. A 25G Quincke spinal needle was used to conduct a subarachnoid block in the interspace between L3 and L4 or L4 and L5 under rigorous aseptic measures. Intrathecal injection of 3

Ml of 0.5% hyperbaric bupivacaine was performed once the release of CSF fluid had been verified. Patients were promptly turned face down.

## Group E - Epidural anesthesia:

The epidural space was located in the interspace between L3 and L4 using a loss-of-resistance approach and an 18G Tuohy needle under aseptic conditions. A catheter was threaded into the epidural space, which is located 3 to 4 centimetres deep. The patient was given a test dose of 3 ml of lignocaine with adrenaline after the aspiration for blood or cerebrospinal fluid came out negative. To get the appropriate block level, 12-15 ml of 0.5% bupivacaine was given gradually.

## Assessment parameters:

## Hemodynamic monitoring:

Heart rate, systolic blood pressure, diastolic blood pressure and mean arterial pressure were recorded:

[1] Baseline (pre-block)

[2] Every 2 minutes for first 10 minutes

[3] Every 5 minutes until end of surgery

Hypotension (fall in SBP >20% of baseline) was treated with IV fluids and mephentermine. Bradycardia (HR < 50/min) was treated with atropine.

## Block characteristics:

## Sensory block:

[1] Assessed by pinprick method

[2] Time to achieve T10 level recorded

[3] Maximum sensory level noted

## Motor block:

[1] Assessed using Modified Bromage Scale

[2] Time to complete motor block recorded

## Duration of block:

[1] Time from drug injection to regression of sensory block to L1

[2] Time to recovery of motor function

## Intraoperative parameters:

[1] Need for supplemental analgesia or sedation

[2] Intraoperative complications

[3] Need for vasopressor support

## Post-operative assessment:

[1] Duration of analgesia (time to first analgesic request)

[2] Complications such as nausea, vomiting, urinary retention, or post-dural puncture headache

## Statistical analysis:

Statistical software (SPSS version 25) was used to examine the data that was input into Microsoft Excel. Mean ± SD is used to represent continuous variables. Percentages used to represent categorical variables The means were compared using the Student's t-test. For categorical variables, the chi-square test is used. p < 0.05 considered statistically significant

## Results:

A total of 50 patients were included and divided equally into two groups: Group S (Spinal anesthesia): n = 25, Group E (Epidural anesthesia): n = 25. Both groups were comparable with respect to demographic variables and surgical duration. Both groups were statistically comparable in age, sex distribution, weight and duration of surgery (p > 0.05). This indicates that subsequent comparisons are unlikely to be influenced by demographic bias (Table 1). Hypotension occurred in 40% of spinal patients compared to 16% in epidural patients, which was statistically significant (p = 0.048). Bradycardia was also more frequent in the spinal group (28% vs 8%, p = 0.041). These findings indicate that epidural anesthesia provided better hemodynamic stability than spinal anesthesia (Table 2). Spinal anesthesia showed significantly faster onset of both sensory and motor block compared to epidural anesthesia (p < 0.001). However, epidural anesthesia provided significantly longer duration of both sensory and motor blockade, suggesting its usefulness in prolonged surgeries and post-operative analgesia. Epidural anesthesia provided nearly double the duration of post-operative analgesia compared to spinal anesthesia (p < 0.001) (Table 3). More than half of the spinal group required rescue analgesia within 4 hours (56% vs 20%, p = 0.009). Post-operative nausea and headache were slightly more frequent in the spinal group, through these differences were not statistically significant (Table 4).

## Discussion:

Fifty patients having orthopedic surgery on their lower limbs were the subjects of this prospective comparative research that compared spinal and epidural anesthesia, focusing on hemodynamic stability and block duration. The results demonstrated that spinal anesthesia provided faster onset and denser block, whereas epidural anesthesia offered superior hemodynamic stability and prolonged analgesia. Hypotension occurred in 40% of participants in the spinal group compared to 16% in the epidural group in the present research. The percentage of patients with bradycardia was 28% in the spinal group and 8% in the epidural group. These findings indicate that epidural anesthesia provided better cardiovascular stability. Spinal anesthesia causes rapid sympathetic blockade due to intrathecal drug spread, leading to sudden vasodilation and reduced venous return. Epidural anesthesia, on the other hand, produces a gradual sympathetic block because the drug diffuses slowly through the dura. Previous studies have reported comparable findings. Hakim *et al.* observed hypotension in nearly 33-40% of spinal anesthesia cases, while epidural techniques showed significantly lower incidence due to slower onset of sympathetic blockade [11]. Similarly, Hewson *et al.* reported that epidural anesthesia resulted in smoother hemodynamic transitions with fewer vasopressor requirements than spinal anesthesia, supporting our findings [12]. The mean onset of sensory block in the present study was significantly faster in the spinal group (approximately 4-5 minutes) compared with the epidural group (12-15 minutes). Motor block onset showed similar differences. This rapid onset with spinal anesthesia is consistent with prior research. Misra *et al.* reported that sequential combined spinal epidural block maintains hemodynamic stability with minimal complications as compared to spinal anaesthesia [13]. Epidural anesthesia requires diffusion across the dura, explaining its slower onset. Clinical studies in orthopedic anesthesia have shown that spinal anesthesia achieves surgical readiness almost 60-70% faster than epidural anesthesia, which correlates with our results [14]. Despite slower onset, epidural anesthesia in our study produced significantly longer sensory and motor block duration. Sensory block lasted approximately 50% longer in the epidural group compared with spinal anesthesia. Post-operative analgesia duration was nearly two times longer with epidural anesthesia. These findings are supported by earlier research demonstrating that continuous epidural dosing prolongs analgesia and reduces early post-operative pain scores [15]. Epidural catheter use also allows extension of anesthesia in prolonged orthopedic procedures, which spinal anesthesia cannot provide once the drug effect declines. Within four hours, 56% of patients in the spinal group needed rescue analgesia, whereas only 20% of patients in the epidural group did so. This difference reflects the limited duration of single-shot spinal anesthesia. Previous trials have shown that epidural anesthesia significantly improves post-operative pain control and reduces systemic opioid requirement by 30-50%, which enhances recovery and patient comfort [16]. The results suggest that spinal anesthesia is ideal for short orthopedic procedures, situations requiring rapid onset and resource-limited settings. Epidural anesthesia may be preferable for Elderly or cardiovascular-compromised patients, longer surgical procedures and Cases requiring prolonged post-operative analgesia. Thus, anesthetic selection should be individualized based on patient condition, surgical duration and post-operative analgesic needs. The limitations included moderate sample size, single-center study and post-operative pain beyond 24 hours not evaluated. Future multicenter trials with larger samples and cost-analysis may further clarify optimal anesthetic choice.

## Conclusion:

Spinal anesthesia provides rapid onset, dense neural block and reliable surgical conditions, making it suitable for shorter procedures. The advantages of epidural anesthesia include better management of post-operative pain, longer sensory and motor blocks and improved hemodynamic stability. It is therefore preferable for prolonged surgeries and patients where cardiovascular stability is important.

## Figures and Tables

**Table 1:** Demographic profile of patients

**Parameter**	**Group S (n=25)**	**Group E (n=25)**	**p value**
Mean age (years)	42.8 ± 11.2	44.1 ± 10.6	0.64
Male (%)	16 (64%)	15 (60%)	0.77
Female (%)	9 (36%)	10 (40%)	0.77
Mean weight (kg)	66.3 ± 8.4	67.8 ± 9.1	0.53
Mean surgery duration (min)	96 ± 18	102 ± 21	0.28

**Table 2:** Hemodynamic changes (incidence of hypotension & bradycardia)

**Parameter**	**Group S (n=25)**	**Group E (n=25)**	**p value**
Hypotension	10 (40%)	4 (16%)	0.048*
Bradycardia	7 (28%)	2 (8%)	0.041*
Vasopressor use	9 (36%)	3 (12%)	0.032*

**Table 3:** Block characteristics

**Parameter**	**Group S**	**Group E**	**p value**
Onset of sensory block (min)	4.2 ± 1.1	12.6 ± 2.3	<0.001*
Onset of motor block (min)	5.1 ± 1.3	15.4 ± 3.1	<0.001*
Duration of sensory block (min)	148 ± 22	224 ± 34	<0.001*
Duration of motor block (min)	132 ± 19	210 ± 28	<0.001*

**Table 4:** Post-operative analgesia and complications

**Parameter**	**Group S**	**Group E**	**p value**
Duration of analgesia (min)	160 ± 24	310 ± 45	<0.001*
Need for rescue analgesia within 4 hrs	14 (56%)	5 (20%)	0.009*
Nausea/Vomiting	6 (24%)	3 (12%)	0.27
Post-dural puncture headache	2 (8%)	0 (0%)	0.15
